# Inhibitory effects of polysorbate 80 on MRSA biofilm formed on different substrates including dermal tissue

**DOI:** 10.1038/s41598-019-39997-3

**Published:** 2019-02-28

**Authors:** Yutaka Ueda, Kota Mashima, Motoyasu Miyazaki, Shuuji Hara, Tohru Takata, Hidetoshi Kamimura, Satoshi Takagi, Shiro Jimi

**Affiliations:** 1 Department of Pharmacy, Fukuoka University Hospital, Fukuoka, Japan; 2 Department of Pharmacy, Fukuoka University Chikushi Hospital, Fukuoka, Japan; 3 Department of Drug Informatics, Faculty of Pharmaceutical Sciences, Fukuoka University, Fukuoka, Japan; 4 Department of Oncology, Hematology and Infectious Diseases, Faculty of Medicine, Fukuoka University, Fukuoka, Japan; 5 Department of Clinical Pharmacology, Faculty of Pharmaceutical Sciences, Fukuoka University, Fukuoka, Japan; 6 Department of Plastic, Reconstructive and Aesthetic Surgery, Faculty of Medicine, Fukuoka University, Fukuoka, Japan; 7 Central Laboratory for Pathology and Morphology, Faculty of Medicine, Fukuoka University, Fukuoka, Japan

## Abstract

Methicillin-resistant *Staphylococcus aureus* (MRSA) forms biofilms on necrotic tissues and medical devices, and causes persistent infections. Surfactants act on biofilms, but their mode of action is still unknown. If used in the clinic, cytotoxicity in tissues should be minimized. In this study, we investigated the inhibitory effect of four different surfactants on MRSA biofilm formation, and found that a nonionic surfactant, polysorbate 80 (PS80), was the most suitable. The biofilm inhibitory effects resulted from the inhibition of bacterial adhesion to substrates rather than biofilm disruption, and the effective dose was less cytotoxic for 3T3 fibroblasts. However, the effects were substrate-dependent: positive for plastic, silicon, and dermal tissues, but negative for stainless-steel. These results indicate that PS80 is effective for prevention of biofilms formed by MRSA on tissues and foreign bodies. Therefore, PS80 could be used in medical practice as a washing solution for wounds and/or pretreatment of indwelling catheters.

## Introduction

*Staphylococcus aureus* (*S. aureus*), an indigenous bacterium, is commonly found in the pores of the skin and nose cavities of healthy people. However, serious infectious disease sometimes occurs if the bacteria invade the epidermal border in patients suffering from skin ulcers and injuries. *S. aureus* acquires drug resistance to commonly prescribed antimicrobial agents in hospitals, due to which methicillin-resistant *S. aureus* (MRSA) has gradually increased in number from the 1960 s^1^; at present, MRSA found in medical practice has become a dominant type of *S. aureus* infection.

MRSA is a biofilm-forming microbe. The biofilm itself exhibits drug tolerance to broad spectrum antibiotics. Biofilms are formed on indwelling foreign bodies including catheters and on necrotic tissues in wounds^2–4^. The drug tolerance property of bacteria increases to several hundred times in biofilms^5,6^, which is explained by decreased drug permeability, appearance of persisters, and intracellular survival^7–9^. Antimicrobial agents thus become ineffective at killing the bacteria in biofilms, leading to a persistent infection. Practical new strategies to eradicate biofilms or inhibit biofilm formation are thus urgently needed.

In the process of biofilm development, bacteria initially attach weakly to substrates, and thereafter, their attachment becomes rigid via the action of polymer molecules including extracellular polysaccharides (EPS)^10^ produced by themselves and attachment proteins including trimeric autotransporter adhesin^11,12^ in gram-negative bacteria, or serine-rich repeat protein^13,14^ and microbial surface components recognizing adhesive matrix molecules (MSCRAMMs)^15,16^ in gram-positive bacteria including *S. aureus*. These factors are crucial for attachment with extracellular matrix components in tissues^17^, including collagen, fibronectin, and vitronectin. On the other hand, bacterial attachment to abiotic indwelling materials involves other mechanisms^15,16,18^: direct molecular interactions, such as electrostatic forces and/or indirect reactions after attachment to biomolecules such as fibrin.

Surfactants are a compound to lower the interface tension between the substances, and act as detergents, wetting agents, forming agents and dispersants. Characteristics of surfactants are generally known to be amphiphilic, due to composing hydrophobic and hydrophilic moiety^19^, and they are classified as cationic, anionic, amphoteric and non-ionic surfactants. Surfactants have long been used in the cosmetic and food industries as well as in the medical field as cleansing and/or bactericidal solutions. We therefore hypothesized that surfactants can disrupt biofilms themselves and/or perturb the process of their formation. This study thus aimed to explore the mechanism of action underlying the inhibition of biofilm formation by MRSA using four different types of surfactants, and to determine the optimization conditions and highly potential surfactants for clinical use.

## Results

### Cell viability reduction by different surfactants

**Figure 1:**
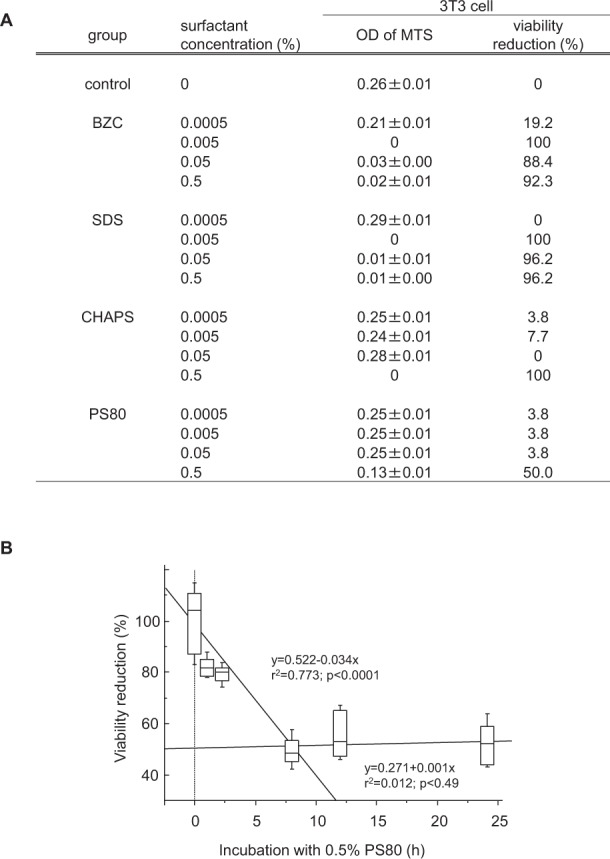
Effect of different surfactants on 3T3 fibroblast viability. (**A**) Different types of surfactants at concentrations up to 0.5% were added to 3T3 cultures in 96 well plates. After incubation for 24 h at 37 °C, MTS viability assay was performed. Values are viability reduction rates (%) against the vehicle control. Although all surfactants in this study were used at less than MTS_50_ cytotoxic doses, PS80 showed the mildest effect in the range. Data are presented as mean ± SE. Sample number/group = 8, duplicated. (**B**) Using 0.5% PS80 in the culture, effects of exposure time on 3T3 cell viability were examined. Cell viability was reduced to about 50% in the first 8 h of incubation, however, it was not further aggravated during 24 h of incubation. Sample number/group = 16, duplicated.

The effects of surfactants on cultured 3T3 cells were examined. Surfactants at concentrations up to 0.5% were added to cultures in 96-well plates, and incubated for 24 h. At the surfactant doses used in the study, all the surfactants showed less than MTS_50_ cytotoxic values. BZC, SDS, and CHAPS showed a precipitous toxic effect (Fig. 1A), whereas PS80 induced the mildest effect. Since 0.5% PS80 induced only 50% viability reduction, the effect of exposure time was further examined. Cell viability was initially reduced to approximately 50% in the first 8 h of incubation; thereafter, it was no longer aggravated and remained unchanged up to 24 h of incubation (Fig. 1B).

### Effects of surfactants on biofilm formation in a plastic tube

**Figure 2:**
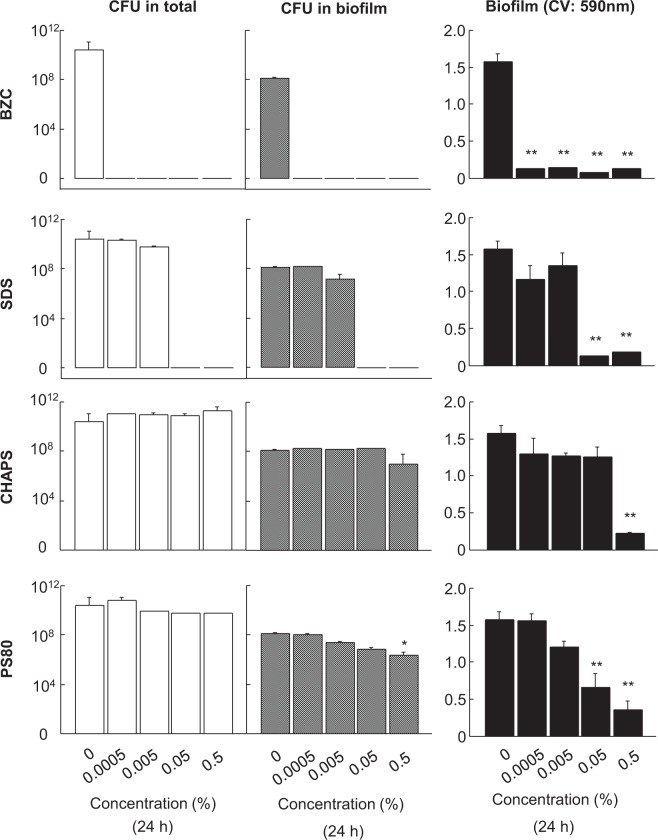
Effects of different surfactants on CFU and biofilm formation values of ATCC BAA-2856 cultured in plastic tubes. Different types of surfactants at concentrations up to 0.5% were added to plastic tubes containing a 1000-fold diluted bacterial solution of ATCC BAA-2856, a biofilm forming MRSA at OD = 0.57, and was incubated for 24 h at 37 °C. After incubation, total bacteria in the tube and in the biofilm formed on the tube surface were assessed using CFU analysis. Biofilm mass was also measured by CV staining. Data are presented as mean ± SE, *p < 0.05, **p < 0.01 vs. vehicle control. Sample number/group = 3, triplicated.

Different surfactants at concentrations up to 0.5% were added during biofilm formation by ATCC BAA-2856 in plastic tubes, and were incubated for 24 h at 37 °C. BZC and SDS induced a strong decline in total CFU values accompanied with a decrease in biofilm formation at concentrations greater than 0.0005% and 0.05%, respectively (Fig. 2). Without a decline in the total CFU, significant decreases in biofilm formation were noted with CHAPS and PS80. However, a significant reduction in both of biofilm and CFU values in the biofilm was only noted with 0.5% PS80.

### Bacterial attachment on a plastic substrate using biofilm forming and low-biofilm forming MRSA

**Figure 3:**
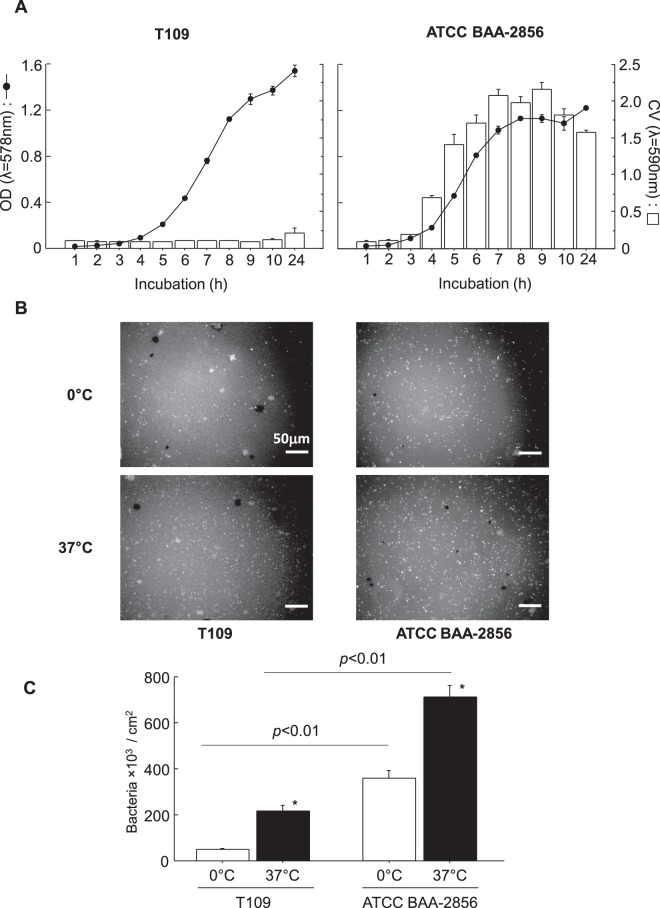
Time courses of bacterial growth and biofilm formation for low and high biofilm formers in plastic tubes and bacterial attachment on a plastic substrate at 0 °C and 37 °C. (**A**) T109 and ATCC BAA-2856 were used for the study. Bacteria were sparsely seeded in plastic tubes, and cultured for 24 h. Bacterial growth was assessed as medial OD values (solid line), and biofilm mass was measured with CV staining (open column). The results clearly showed that the bacteria were low and high biofilm-forming MRSA, respectively. Data: mean ± SE. Sample number/group = 3, quadruplicated. (**B**) Plastic chips (1 × 1 cm pieces of OHP sheet) were immersed in confluent bacterial solutions, and incubated at 0 °C and 37 °C for 1 h. After incubation, bacteria adhered on the surface were visualized using FITC-labeled *S. aureus* antibody. Bar = 50 μm. (**C**) Bacteria adhered on plastic chips were counted using the morphometric method as described in the Materials and Methods. Data are presented as bacteria number/cm^2^ area, mean ± SE, *p < 0.01 vs. 0 °C. Sample number/group ≥9, duplicated.

To determine the relationship between bacterial attachment to substrates and biofilm-forming ability, a low biofilm-forming MRSA (T109) strain was also used. The bacterial growth curves and biofilm formation in T109 and ATCC BAA-2856 are shown in Fig. 3A, indicating that no difference was found in their growth, but their biofilm-forming ability was significantly different (8 times different at 24 h). To determine their attachment ability on a plastic substrate, OHP sheets were immersed in a confluent planktonic bacterial solution at 0 °C or 37 °C for 1 h. Bacteria attached to the OHP sheet are shown in Fig. 3B, and their number is shown in Fig. 3C. At 0 °C and 37 °C, the attached cell number was significantly greater in ATCC BAA-2856 than in T109: approximately 7 times greater at 0 °C (p < 0.01) and about 3 times greater at 37 °C (p < 0.01). In both bacteria, the attached cell number was increased with an increase in temperature.

### MRSA attachment inhibition by surfactants

CHAPS and PS80 were chosen as surfactants inducing an anti-biofilm effect not due to bactericidal effects. The effects of 0.5% PS80 or 0.5% CHAPS were tested on planktonic bacterial attachment to an OHP plastic substrate using ATCC BAA-2856 and T109. Both surfactants were effective for MRSA attachment inhibition to the substrate (Table 1), but PS80 was more effective regardless of the MRSA type and temperature.

**Table 1:** Effect of CHAPS and PS80 on the attachment of low and high biofilm formers to a plastic substrate.

bacteria	cultured at	control	bacteria × 10^3^/cm^2^ plastic chips			
			0.5% CHAPS	reduction (%)	0.5% PS80	reduction (%)
T109	0 °C	49.0 ± 5.4	30.7 ± 10.8	37	15.7 ± 4.1**	68
	37 °C	215.8 ± 24.5	114.2 ± 17.1*	47	21.6 ± 3.5**	90
ATCC BAA-2856	0 °C	360.1 ± 31.2	223.6 ± 50.3*	38	15.2 ± 3.8**	96
	37 °C	710.6 ± 51.2	142.8 ± 7.3**	80	23.4 ± 6.1**	97

T109, a low biofilm-forming MRSA, and ATCC BAA-2856, a high biofilm-forming MRSA, were used. Plastic chips (1 × 1 cm pieces of OHP sheet) were immersed in confluent bacterial solutions containing 0.5% PS80 or 0.5% CHAPS, both of which exerted biofilm inhibitory effects as shown in Fig. 2. They were incubated at 0 °C or 37 °C for 1 h. After incubation, the bacteria adhered on the chip were visualized using FITC-labeled *S. aureus* antibody, and their number was counted by a morphometrical method as described in the Materials and Methods. Data are presented as number of bacteria/cm^2^ area, mean ± SE, *p < 0.05 and **p < 0.01 vs. control in each temperature. Sample number/group ≥8, duplicated.

### Biofilm inhibition by PS80 during biofilm development

**Figure 4:**
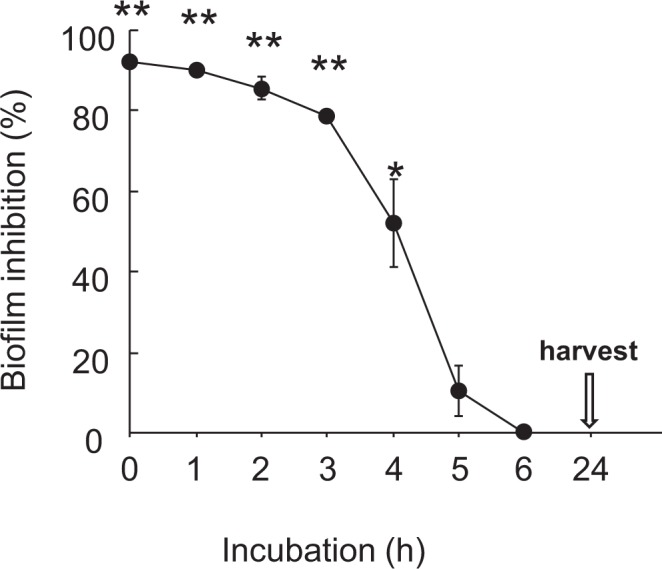
Inhibitory effects of 0.5% PS80 addition at different time points on biofilm formation during 24 h incubation. During the 24 h biofilm assay starting from 1000-fold diluted ATCC BAA-2856 bacterial solution at OD = 0.57, 0.5% PS80 was added at 0, 1, 2, 3, 4, 5, and 6 h of incubation. At the end of the study, the biofilm mass formed on the tube wall surface was measured with CV stain. Data are presented as mean ± SE. Sample number/group = 3, triplicated.

During biofilm development in the ATCC BAA-2856 culture for 24 h, 0.5% PS80 was added at 0, 1, 2, 3, 4, 5, and 6 h of incubation. The inhibitory effect of 0.5% PS80 on the biofilm was only found at additions as early as 4 h after incubation (Fig. 4), but its effect was subsequently vanished. This indicates that PS80 may work only in the premature phase of biofilm development, including attachment, rather than in the maturation phase of the biofilm.

### Inhibitory effects of PS80 on bacterial attachment to dermal tissues

**Figure 5:**
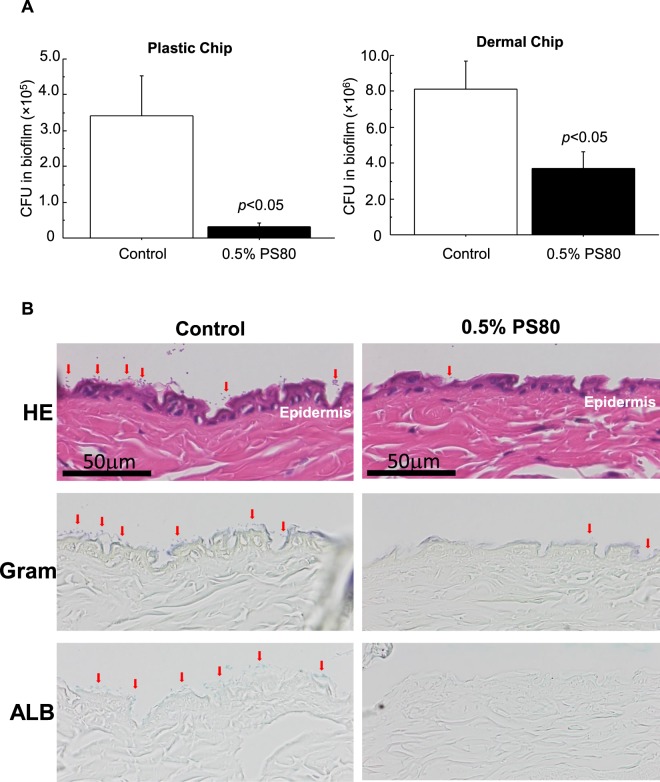
Inhibitory effects of 0.5% PS80 on bacterial attachment to dermal and plastic chips. (**A**) Plastic chips (1 × 1 cm pieces of OHP sheet) and dermal chip (1 × 1 cm pieces of mouse skin) were immersed in confluent bacterial solutions with/without 0.5% PS80, and incubated at 37 °C for 1 h. After incubation, bacteria adhered on the chips were assessed by CFU analysis. Data are presented as mean ± SE. Sample number/group = 5, duplicated. (**B**) Serial sections of dermal chips were subjected to HE, Gram and ALB stainings after incubation in confluent bacterial solutions. Gram-positive bacteria attached on the surface show a fine spherical structure (red arrows) and ALB-positive EPS containing biofilm (red arrows). Addition of 0.5% PS80 apparently decreased the number of attached bacteria accompanied with biofilm on the dermal tissue.

Dermal chips prepared from mouse skin tissue and OHP plastic chips were utilized as substrates. Inhibitory effects of 0.5% PS80 on MRSA attachment were examined by CFU analysis and morphology (Fig. 5). In the CFU analysis, 10 times more bacteria were attached to the dermal tissue compared to the plastic substrate (Fig. 5A), and PS80 effectively inhibited their attachment to both the dermal and plastic chips with 46% and 91% reduction, respectively. When the dermal tissue was microscopically observed, no gram-positive indigenous bacteria were found in a dermal tissue without MRSA exposure. After MRSA exposure, gram-positive bacteria were attached on the surface, showing a fine spherical structure (red arrows: Fig. 5B), and ALB-positive EPS was also found around bacteria on the epidermis (red arrows: Fig. 5B). Addition of 0.5% PS80 apparently decreased the attached bacteria and ALB-positive spots in number on the dermal tissue.

### Effects of PS80 on biofilm formation on silicon and stainless steel

**Figure 6:**
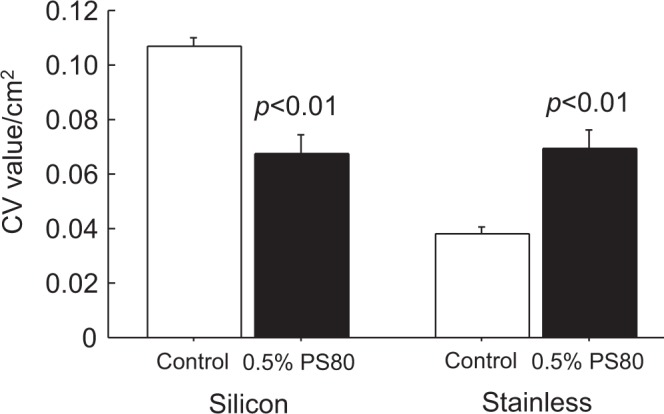
Inhibitory effects of 0.5% PS80 on biofilm formation with silicon and stainless steel substrates. Diluted ATCC BAA-2856 bacterial solutions (1000-fold from OD = 0.57) with/without 0.5% PS80 were added to silicon-coated tubes and incubated for 24 h at 37 °C. Stainless steel sheets cut to 3 × 3 cm were also immersed in bacterial solutions with/without 0.5% PS80 for 24 h at 37 °C. After incubation, the biofilm mass formed on the silicon substrate and stainless steel substrate was measured by CV staining. Data are presented as CV values/cm^2^, mean ± SE. Sample number/group = 10, triplicated.

Similarly, silicon and stainless steel were adopted as substrates, and the inhibitory effects of 0.5% PS80 on biofilm formation were examined (Fig. 6). PS80 significantly suppressed biofilm formation on the silicon substrate, whereas biofilm formation on the stainless steel substrate was accelerated.

## Discussion

Till date, there have been no studies exploring the effects of surfactants on MRSA biofilm development. The present study was therefore conducted to explore surfactants with anti-biofilm effects, such as inhibition of biofilm development or bacterial killing, under physiologically safe conditions. We therefore performed a MRSA biofilm assay (mass and CFU) and 3T3 cell viability assay using different types of surfactants at a wide range of concentrations (0.0005–0.5%). Inhibition of biofilm formation arose from bacterial killing or inhibition of attachment to substrates without bacterial killing (Summary of the results is shown in Table 2); the former was found with BZC and SDS, and the latter was found with CHAPS and PS80. Cytotoxic effects on 3T3 cells were found with BZC, SDS, and CHAPS, but not with PS80. Thus, PS80 could be used as a surfactant to inhibit biofilm formation in medical practice.

**Table 2:** Summary: Effects of surfactants.

surfactant	concentration (%)	MRSA (ATCC BAA-2856)			3T3 cell
		inhibition of biofilm formation	growth inhibiton	toxicity	toxicity
BZC	0.0005	+	+	−	±
	0.005	+	+	−	+
	0.05	+	+	−	+
	0.5	+	+	+	+
SDS	0.0005	−	−	−	−
	0.005	−	−	−	+
	0.05	+	+	−	+
	0.5	+	+	−	+
CHAPS	0.0005	−	−	−	−
	0.005	−	−	−	−
	0.05	−	−	−	−
	0.5	+	−	−	+
PS80	0.0005	−	−	−	−
	0.005	−	−	−	−
	0.05	+	−	−	−
	0.5	+	−	−	±

The bactericidal action and toxicity of different surfactants used is summarized. All effects were graded into three excluding “toxicity” in bacteria (total negative: −, positive: +) using CFU analysis. Toxicity (%-viability reduction) in 3T3 fibroblasts was as follows: 10% ≥: −, 11–50%: ±, 51% ≤: +.

Bacterial adherence to substrates is an important initial step prior to biofilm development. The mechanism of bacterial attachment to abiotic materials is thought to involve nonspecific attachments regulated by the hydrophobicity, hydrophilicity, or charge of the material^15,18^. EPS, teichoic acid, and extracellular DNA, known as bacterial extracellular matrix components/scaffolds, also act as a glue for mutual bacterial binding, therefore promoting adhesion and aggregation^18^. For plastic substrates, covalently-linked surface proteins of the MSCRAMM family, Bap and SasC have been utilized for their attachment^20,21^. They have also attached to our tissue constituents using fibronectin-binding proteins and teichoic acid^17^. In this study, bacteria were chosen in terms of biofilm-forming ability; one was the highest biofilm former (ATCC BAA-2856) and the other was the lowest biofilm former (T109) among our 173 clinical isolates. We then checked their attachment ability in ice, a biological action-free condition. ATCC BAA-2856 showed greater attachment ability to a plastic substrate compared to T109, suggesting that some components present in biofilm construction may be involved in the attachment between bacteria and plastic.

The mechanisms of biofilm development involve two distinctive steps: cellular attachment to substrates^22,23^, as an initial step, and proliferation and matrix production on substrates, as a progression step. To explore the initial step, ATCC BAA-2856 and T109 were incubated on ice or at 37 °C in the presence/absence of CHAPS or PS80. In the absence of surfactants, ATCC BAA-2856 showed stronger attachment activity than T109 (approximately 3 times at 37 °C and approximately 7 times on ice), and low temperature reduced the attachment to a greater extent in ATCC BAA-2856 (approximately half) than in T109 (about 1/5). Such cellular attachments in both cells were strongly repressed by PS80 both in ice and at 37 °C and by CHAPS at 37 °C. The results clearly demonstrate that biofilm formers possessed strong attachment activity to plastic substrates even under conditions of suppressed-biological activity in ice. PS80 is therefore able to inhibit microbial attachment to substrates via a physical action. The possible mechanism underlying this phenomenon is the action of surfactants primarily on either lipophilic cellular membranes or the substrates themselves, or both. We thus performed an additional experiment using the LIVE/DEAD assay; however, no distinct effects of PS80 on the cellular membrane were noted (Fig. S1). Thus, the effects of PS80 on substrates are the most likely explanation for the PS80-mediated inhibition of biofilm formation.

We next investigated the inhibitory effects of PS80 on biofilm development. Biofilm formation was effectively prohibited by an early treatment within 4 h of incubation, whereas perfect inhibition was noted with simultaneous addition at the time of cell seeding (0 h). At 5 h of incubation, the biofilm entered the maturation phase (Fig. 3A), and none of the inhibitory effects were observed. These results also indicate that PS80 acts only in the premature phase of biofilm formation, including bacterial attachment. Similar lines of evidence have been reported by Sloup *et al*.^24^ using *Escherichia coli* O104:H4 biofilms.

The final aim of this study was the usage of surfactants in medical practice to prevent biofilm formation. Biofilms are frequently developed in ulcerative wounded tissues. We thus examined the tissue substrate using a novel mouse dermal chip, which was established in our previous study^25^. Compared to the plastic substrate, MRSA showed greater attachment to the dermal tissue surface, and PS80 prevented bacterial attachment to plastic as well as dermal tissue. Biofilms are also developed on indwelling catheters and metals including stainless streel and titanium^23^. We thus chose silicon and stainless streel as biofilm substrates. Interestingly, PS80 prevented biofilm formation only on the silicon substrate but not on stainless steel. As silicon, unlike stainless steel, is a hydrophobic material, the nonionic PS80 can access the surface, by which bacterial adhesion can be suppressed.

In the four different types of surfactants tested in this study, inhibition of biofilm formation accompanied with bactericidal effects was noted with BZC and SDS, but these cannot be used because of their high toxicity to tissue cells. In contrast, PS80 is well tolerated and used as a surfactant and emulsifier in the cosmetic, food, and pharmaceutical industries at a concentration range from 0.1% to over 1%^26^. In the present study, the effective dose of PS80 was greater than 0.05%, which is far lower than that used in the industry. In addition, PS80 has been designated as a Generally Recognized as Safe (GRAS) compound by the Food and Drug Administration (FDA)^27^.

In conclusion, the effects of different types of surfactants on biofilm formation by MRSA were determined. We clearly showed that PS80 effectively inhibits biofilm formation on different substrates such as plastic, silicon, and dermal tissue. To the best of our knowledge, this study is first to address the mechanisms underlying the effect of PS80 on MRSA biofilm formation. PS80 is well tolerated, and can be added to washing solutions for ulcerative wounded tissues and indwelling materials, as well as to intravenous infusion fluids to prevent biofilm formation.

## Methods

### Surfactants

Four different types of surfactants were used: Benzalkonium chloride (BZC) (Sigma-Aldrich, Tokyo, Japan) as a cationic surfactant, sodium dodecyl sulfate (SDS) (Sigma-Aldrich) as an anionic surfactant, 3-(3-Cholamidepropyl) dimethylammonio-1-propane-sulphonate (CHAPS) (Sigma-Aldrich) as an amphoteric surfactant, and Polysorbate 80 (PS80) (Sigma-Aldrich) as a non-ionic surfactant. Surfactants dissolved in Tryptic Soy Broth (TSB) were serially diluted 10-fold and final concentrations of the surfactants were 0.0005%, 0.005%, 0.05%, and 0.5%.

### Cell culture

Mouse embryonic 3T3 fibroblasts were purchased from the American Type Culture Collection (ATCC) (Manassas, VA, USA) and were grown in Dulbecco’s modified Eagle’s medium (DMEM) (Thermo Fisher Scientific K.K., Tokyo, Japan) containing 10% fetal bovine serum (NICHIREI BIOSCIENCES INC., Tokyo, Japan) and 1% penicillin-streptomycin (Thermo Fisher Scientific K.K.) at 37 °C under a humidified atmosphere of 5% CO_2_/95% air.

### Cell viability assay

Different concentrations of surfactants added to 3T3 cells (3 × 10^3^/well of 96-well plate) were incubated for 24 h at 37 °C. After incubation, 20 µL of CellTiter 96 AQueous One Solution Reagent (Promega Co., Madison, WI, USA), which contains a tetrazolium compound 3-(4,5-dimethyl-2-yl)-5-(3-carboxymethoxyphenyl)-2-(4-sulfophenyl)-2H-tetrazolium, inner salt (MTS), was added to each well. After incubation for 1 h, colorimetric absorbance in each well was measured at 490 nm using a microplate reader (Sunrise Rainbow: Tecan Japan Co., Ltd., Kanagawa, Japan). Viability reduction rates (%) in each compound were calculated against the mean value of the vehicle control. In some experiments, 0.5% PS80 was added to cultures at 0, 1, 2, 8, and 12 h during the 24 h incubation, and viability was assessed at the end of incubation.

### Bacteria

A clinical strain of MRSA, namely ATCC BAA-2856^28,29^ (OJ1), designated as a high-biofilm former and a low-biofilm forming MRSA isolate, namely T109^30^ were used in this study; both these isolates were established in our previous studies. The bacteria were thawed from the stocks and incubated on Tryptic Soy Agar (TSA) (Becton, Dickinson and Company, Franklin Lakes, NJ, USA) containing 0.5% NaCl. When the colonies formed, one colony was collected and inoculated in 5 mL of TSB (Becton, Dickinson and Company) in 12 mL plastic test tubes with screw caps (SARSTEDT, Tokyo, Japan), and cultured at 37 °C until the optical density (OD) reached 0.57. Subsequently, a 1000-fold diluted bacterial solution in TSB was generally used for bacterial growth and biofilm analysis unless otherwise indicated.

### Bacterial growth, biofilm formation, and colony forming analysis

Bacterial growth in a 12-mL plastic tube was assessed by turbidity increment in media at 578 nm in an absorption spectrometer (GENESYS 10S VIS, Thermo Scientific, LMS, Tokyo, Japan) after stirring by inversion mixing. The turbidity was used as a bacterial growth index. The biofilm attached on the surface of tubes was obtained after washing 3 times with PBS. The amount of biofilm formed after overnight incubation was measured using crystal violet (CV) (Sigma Aldrich) staining according to previously reported methods^31^. Bacteria were harvested from TSA, suspended in test tubes containing 5 mL of TSB, and cultured at 37 °C for 24 h. Floating bacteria were discarded, and the biofilm that was adhering to the wall was washed with PBS, (pH 7.4). Then, 100% ethanol was added, and the samples were dried and subjected to CV staining for 10 min. After staining, the samples were washed in running water until the dye was completely eluted, and the dye that was attached to the surface of the test tubes was dissolved with 3 mL of 30% acetic acid. The absorbance of the dye solution (absorbance wavelength 570 nm) was measured using a spectrophotometer. To determine the number of live bacterial cells, the bacteria in tubes were dissociated using an ultrasonic generator (Sonifier 250: Branson Ultrasonics, Emerson Japan, Ltd., Kanagawa, Japan) at 50% duty cycle and output level 1 for 3 min in ice. Colony-forming units (CFU) in the total bacteria (planktonic and biofilm) and the biofilm alone were then assessed. In some experiments for surfactants’ toxicity, surfactants added into bacterial solution grown in confluent, and 50 μL of the bacteria solution were inoculated on TSA. After 24 h incubation at 37 °C, if colonies were grown, surfactant was determined to be non-cytotoxic, if not, it was to be cytotoxic.

### Effect of surfactants on bacterial attachment to plastic substrates

ATCC BAA-2856 and T109 in TSB were incubated overnight at 37 °C, and confluent bacterial solutions without adherent cells were collected. Bacteria in TSB were ultra-sonicated using Sonifer 250 for 3 min, and OD levels (λ = 578) in ATCC BAA-2856 (about 1.3) and T109 (about 1.6) were adjusted to 1.3 using PBS. Each bacterial culture with/without 0.5% CHAPS and 0.5% PS80 was incubated with a plastic sheet (OHP) cut to 1 × 8 cm pieces (3 M Japan Ltd., Tokyo, Japan) for 1 h in ice or at 37 °C. After incubation, the plastic sheets directly immersed in 5% neutral formalin (pH 7.4) without air-dry and were fixed for 1 h. After washing 3 times with PBS, bacteria adhered on the plastic sheet were incubated with FITC-labeled *S. aureus* antibody (Virostat, Portland, ME) for 1 h at room temperature. Fluorescence signals on the plastic sheet were randomly photographed under a microscope (Biozero, Keyence Co., Osaka, Japan) using the 20× objective lens. FITC-signals on a picture (0.13 mm^2^) were morphometrically analyzed using a software (VH analyzer: Kyence Co.), and the number of positive spots/cm^2^ and each spot area were obtained.

### Effect of PS80 on biofilm development

During biofilm development by ATCC BAA-2856 on a plastic tube surface for 24 h at 37 °C, 0.5% PS80 was added at 0, 1, 2, 3, 4, 5, and 6 h of incubation. The biofilm in tubes was then assessed by CV staining.

### Effects of PS80 on bacterial attachment to dermal and plastic chips

The preparation of mouse dermal chips has been described previously^25^. All animal experiments received prior approval from the animal experiment approval committee of Fukuoka University Animal Center (approval number 1210608). Female C57BL/6N mice (Japan SLC) were used for the study. Under anesthesia with Somunopentyl (Kyoritsu-Seiyaku), depilation was performed using a commercial hair remover. Animals were sacrificed by cervical dislocation and their complete skin tissue was obtained. After removal of excess fat and muscle with tweezers, the internal face of the skin was spread and adhered on a cardboard, and fixed in 99% ethanol for 24 h. The fixed skin was then dried on a clean bench with airflow. The skin sheet on the cardboard was cut into 1 × 1 cm pieces and sterilized using ethylene oxide gas. These pieces, referred to as dermal chips, were stored at 4 °C and used within 3 months. The skin structure was well preserved in the dermal chips^25^. As a control substrate, 1 × 1 cm OHP plastic sheets (3 M Japan Ltd.) were used.

Confluent cultures of ATCC BAA-2856 after overnight incubation were used. Bacterial cultures with/without 0.5% PS80 were incubated with dermal and plastic chips at 37 °C for 1 h. After incubation, the bacteria attached on the dermal and plastic chips were washed 3 times in PBS, ultrasonically dissociated using Sonifer 250 at 50% duty cycle and output level 1 for 3 min in ice, and the obtained bacterial suspension was used for CFU analysis. Dermal chips were fixed in 5% neutral formalin and embedded in paraffin blocks. The blocks were cut into 4 µm thin sections and used for histological staining, i.e. hematoxylin–eosin staining, Gram staining and Alcian blue staining (pH 2.5) (ALB).

### Effect of surfactants on biofilm formation on different substrates

In addition to the plastic substrate, silicon and stainless substrates were also used. ATCC BAA-2856 bacterial solution diluted 1000-fold at OD = 0.75 was poured into silicon-coated tubes (AGC Techno Glass Co. Ltd., Shizuoka, Japan) or plastic tubes with rolled stainless sheets (Taiho Trading Co., Ltd., Tokyo, Japan) cut into 3 × 3 cm pieces, and incubated at 37 °C for 24 h in the presence of different surfactants (SDS, CHAPS, and PS80). After incubation, the biofilms formed on the substrate surfaces were stained with CV, and absorbance of the extracted dye was used as a measure of biofilm formation.

### Ethics approval

All methods involving bacterial handlings and animal treatments were performed in accordance with the relevant guidelines and regulations under the Fukuoka University’s experiment regulations.

### Data and statistical analysis

Results from two different experimental groups were initially compared with distribution analysis using F-test, and then student’s *t*-test or Mann-Whitney U test were performed. P values < 0.05 were considered to denote statistical significance. Similar experiments were repeated at least two times. Data are expressed as mean values ± standard error.

## Supplementary information

Supplemental Figure
