# Supplementary material for: Inhibitory effects of polysorbate 80 on MRSA biofilm formed on different substrates including dermal tissue
Source: Sci Rep. 2019 Feb 28;9:3128. doi: 10.1038/s41598-019-39997-3 (PMC6395670; doi:10.1038/s41598-019-39997-3)
Supplement: Supplementary file 1 — Supplemental Figure [file 41598_2019_39997_MOESM1_ESM.docx]

**Inhibitory effects of polysorbate 80 on MRSA biofilm formed on different substrates including dermal tissue**

Yutaka Ueda^a^, Kota Mashima^a^, Motoyasu Miyazaki^b^, Shuuji Hara^c^, Tohru Takata^d^, Hidetoshi Kamimura^a,e^, Satoshi Takagi^f^, Shiro Jimi^g^

^a^ Department of Pharmacy, Fukuoka University Hospital, Fukuoka, Japan

^b^ Department of Pharmacy, Fukuoka University Chikushi Hospital, Fukuoka, Japan

^c^ Department of Drug Informatics, Faculty of Pharmaceutical Sciences, Fukuoka University, Fukuoka, Japan.

^d^ Department of Oncology, Hematology and Infectious Diseases, Faculty of Medicine, Fukuoka University, Fukuoka, Japan.

^e^ Department of Clinical Pharmacology, Faculty of Pharmaceutical Sciences, Fukuoka University, Fukuoka, Japan.

^f^ Department of Plastic, Reconstructive and Aesthetic Surgery, Faculty of Medicine, Fukuoka University, Fukuoka, Japan.

^g^ Central Laboratory for Pathology and Morphology, Faculty of Medicine, Fukuoka University, Fukuoka, Japan


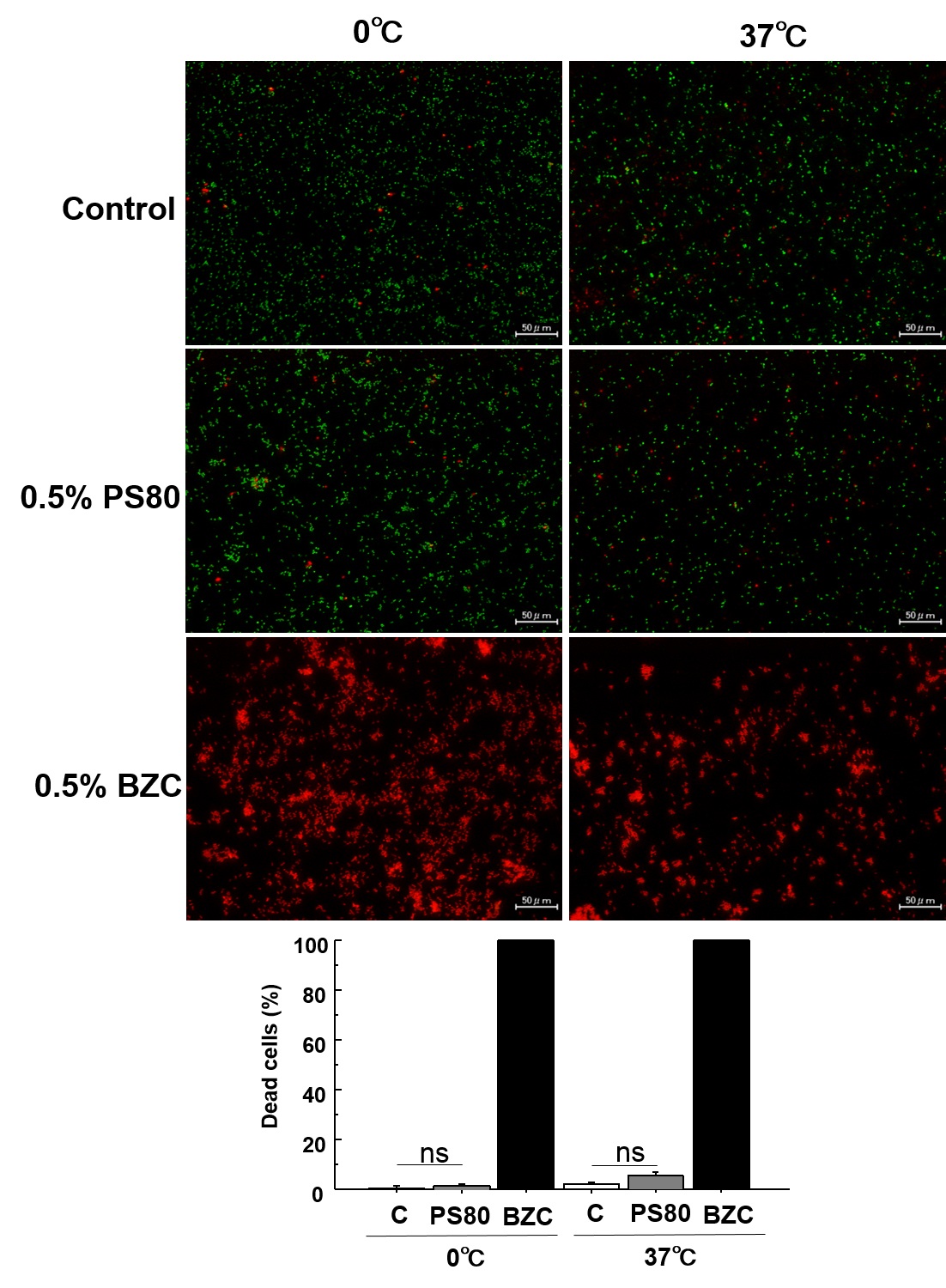


**Supplemental Figure S1: Effects of PS80 on the cell membrane of ATCC BAA-2856**

Bacterial LIVE/DEAD assay, a kit for which was procured from Thermo Fisher Scientific K.K., was performed according to the manufacturer’s protocol. Bacteria were prepared as described for the attachment assay: confluent bacteria in TSB treated with saline (vehicle control), 0.5% BZC, or 0.5% PS80 were incubated at 0ºC or 37ºC for 1 h. After incubation, living cells (green color) and membrane-perturbed dead cells (red color) were identified using fluorescence microscopy.

In both the control and PS80-treated groups, the proportion of dead cells among the cells incubated at 0ºC or 37ºC was quite low (less than 10%), compared with the BZC-treated group (100%). Nevertheless, statistically, the proportions of dead cells in both the groups were significantly higher in case of the cells incubated at 37ºC than those incubated at 0ºC. PS80, however, did not cause bacterial membrane perturbation leading to cell death.

These results indicate that confluent bacteria could die to some extent due to their overgrowth after incubation at 37ºC. However, no significant effects of PS80 on the bacterial membrane were noted.
